# A novel guided approach to radiofrequency ablation of thyroid nodules: the Toronto Sunnybrook experience

**DOI:** 10.3389/fendo.2024.1402605

**Published:** 2024-07-24

**Authors:** Leba Michael Sarkis, Kevin Higgins, Danny Enepekides, Antoine Eskander

**Affiliations:** ^1^ Department of Otolaryngology-Head and Neck Surgery, Sunnybrook Health Sciences Centre, Toronto, ON, Canada; ^2^ Sydney Medical School, University of Sydney, Sydney, NSW, Australia; ^3^ Department of Otolaryngology – Head & Neck Surgery, Michael Garron Hospital, Toronto, ON, Canada; ^4^ Institute of Health Policy, Management, and Evaluation, University of Toronto, Toronto, ON, Canada

**Keywords:** thyroid, nodule, radiofrequency ablation, guide, benign, autonomous

## Abstract

**Introduction:**

Thyroid nodules are extremely common being detected by ultrasonography in up to 67% of the population, with current surgical tenet maintaining that lobectomy is required for large symptomatic benign nodules or autonomously functionally nodules resulting in a risk of hypothyroidism or recurrent laryngeal nerve injury even in high volume centres. The introduction of radiofrequency ablation (RFA) has allowed thermal ablation of both benign and autonomously functioning thyroid nodules with minimal morbidity. The moving shot technique is the most well-established technique in performing RFA of thyroid nodules, and has proven to be safe, efficacious, accurate and successful amongst experienced clinicians. The purpose of this article to propose the use of a novel guide when performing RFA of thyroid nodules in clinical practice utilizing the moving shot technique.

**Methods:**

The technique proposed of RFA involves the use of a 10MHz linear ultrasound probe attached to an 18G guide which provides robust in line visualisation of a 7cm or 10cm radiofrequency probe tip (STARmed, Seoul, Korea) utilizing the trans isthmic moving shot technique. A geometric analysis of the guide has been illustrated diagrammatically.

**Results:**

The use of an 18G radiofrequency probe guide (CIVCO Infiniti Plus™ Needle Guide) maintains in line visualisation of the radiofrequency probe over a cross-sectional area up to 28cm^2^, facilitating efficient and complete ablation of conceptual subunits during RFA of thyroid nodules.

**Discussion:**

Radiofrequency ablation of thyroid nodules can be performed safely and effectively using the novel radiofrequency probe guide proposed which we believe potentially improves both accuracy and overall efficiency, along with operator confidence in maintaining visualisation of the probe tip, and hence we believe provides a valuable addition to the armamentarium of clinicians wishing to embark on performing RFA of thyroid nodules.

## Introduction

Thyroid nodules are common and detectable by ultrasonography in up to 13-67% of individuals (1), with higher frequencies in women and the elderly even when the gland is normal on palpation. The initial work up of thyroid nodules has been well established and includes a thorough clinical history to determine risk factors for malignancy, clinical examination and an assessment of thyroid function followed by ultrasonography of both the thyroid bed and lateral neck. The vast majority do not require treatment, and current treatment paradigm maintains that surgery is required for nodules to confirm either suspicious lesions on fine needle aspiration biopsy (FNA) or on nodules that are confirmed to be malignant. However following FNA confirmation of benign nodules, or in isolated autonomously functioning thyroid nodules (AFTN), the risk of malignancy is low in the order of 2-6%. The majority of solitary toxic nodules represent follicular adenomas with a high prevalence of activating mutations in the TSH receptor and their risk of malignancy is less than 3% (2, 3).

Non-malignant (large benign or autonomously functioning) nodules sometimes, however, do require surgical intervention in the form of partial thyroidectomy (4). This leaves the patient with a small cervical scar and renders up to 22% of patients with hypothyroidism for life (5). Despite advances in surgical training, thyroidectomy still carries with it a 2-5% complication rate, even when performed by high volume surgeons (6), resulting in many patients delaying or avoiding surgery entirely.

With the increasing use of clinician-initiated point of care ultrasonography, ultrasound-guided thermal ablative techniques have been introduced by interventional thyroidologists (surgeons, endocrinologists, and interventional radiologists) to effectively treat and manage benign or AFTN. Radiofrequency ablation (RFA) of the thyroid gland was first introduced in human studies in 2006 (7) and multiple international guidelines (8–13) have since been developed including those most recently released by the American Thyroid Association (ATA) (14). The basic principle of RFA involves using a high-frequency alternating electric current that oscillates resulting in formation of a closed electrical circuit (15). The subsequent activation of radiofrequency power via a probe activates positively charged sodium and potassium ions and negatively charged chloride ions within the adjacent tissue and as they attempt to align with the polarity established by the high frequency electrical current, generate either frictional or resistive heat which is then transmitted and conducted to adjacent tissue (16). This generates heat between 60-100 degrees Celsius around the electrode tip that induces near instantaneous induction of protein coagulation with additional thermal injury via conduction that irreversibly damages cellular enzymes resulting in cell death by coagulative necrosis (15, 16). Perhaps the most established technique in implementing RFA in the thyroid is the moving-shot technique, proposed by Baek et al (17) as a means of maximising the efficiency of frictional heat generated at the electrode tip resulting in a more predictable ablation zone and minimizing risk of damages to adjacent visceral organs and nerves induced by thermal conduction. With this technique, the deepest portion of the nodule is targeted first using a trans-isthmic long-axis technique with a slow withdrawal before the next portion of the nodule is targeted, again from a deep to superficial approach. This technique has a well-established safety profile.

The purpose of this paper is to provide our approach to safely performing RFA for both benign and AFTN using a moving shot trans-isthmic long-axis technique. More specifically, we provide a pictorial and geometric analysis of the benefits and limitations of a novel ultrasound probe needle guide (CIVCO Infiniti Plus™).

## Methods

### Indications

RFA at Sunnybrook Health Sciences Centre is performed under ultrasound guidance by a clinician with experience in performing ultrasonography, FNA biopsies of the thyroid gland as well as core biopsies of neck masses. Indications for RFA are in keeping with the most recent ATA consensus statement, namely two thyroid FNA confirmed benign biopsies or AFTN (14).

### Procedure preparation

The patient is placed in a supine position with the head elevated at approximately 30 degrees. Grounding pads are then applied to the patient’s upper thigh to establish a complete grounded electrical circuit by also connecting them to the radiofrequency device. The generator is subsequently connected to a straight monopolar RF electrode with an active tip length between 5, 7 or 10mm, depending on the size of the nodule (STARmed Seoul, Korea), and a 7cm RF probe is most commonly employed. A cooling pump is used to circulate saline through the electrode and lower the temperature around the active tip to maximize ablative margin whilst minimizing tissue charring. Local anaesthetic being lidocaine with 1:100,000 adrenaline is injected around the skin at the site of entry of the probe for the trans-isthmic approach followed by a superficial cervical plexus block. Additional anaesthetic and 5% dextrose is then injected under ultrasound guidance using a spinal needle in a pericapsular plane producing a visualised anechoic band that hydrodissects and separates the nodule from the infrahyoid musculature and laterally away from the carotid sheath and a potentially medialized vagus nerve.

### Guide technique

**Figure 1:**
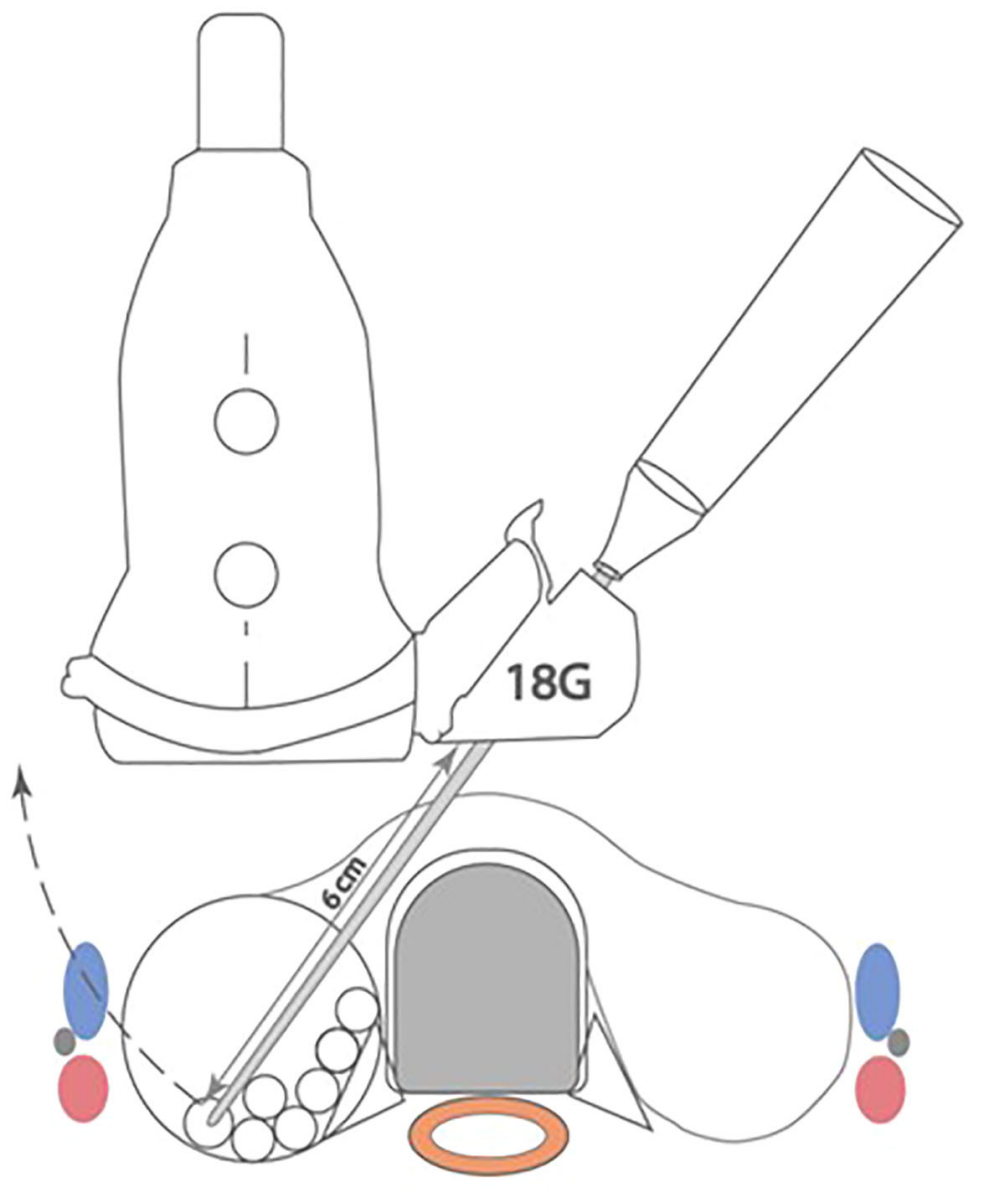
Radiofrequency probe with guide attached demonstrating in line stabilization and visualization during procedure.

**Figure 2:**
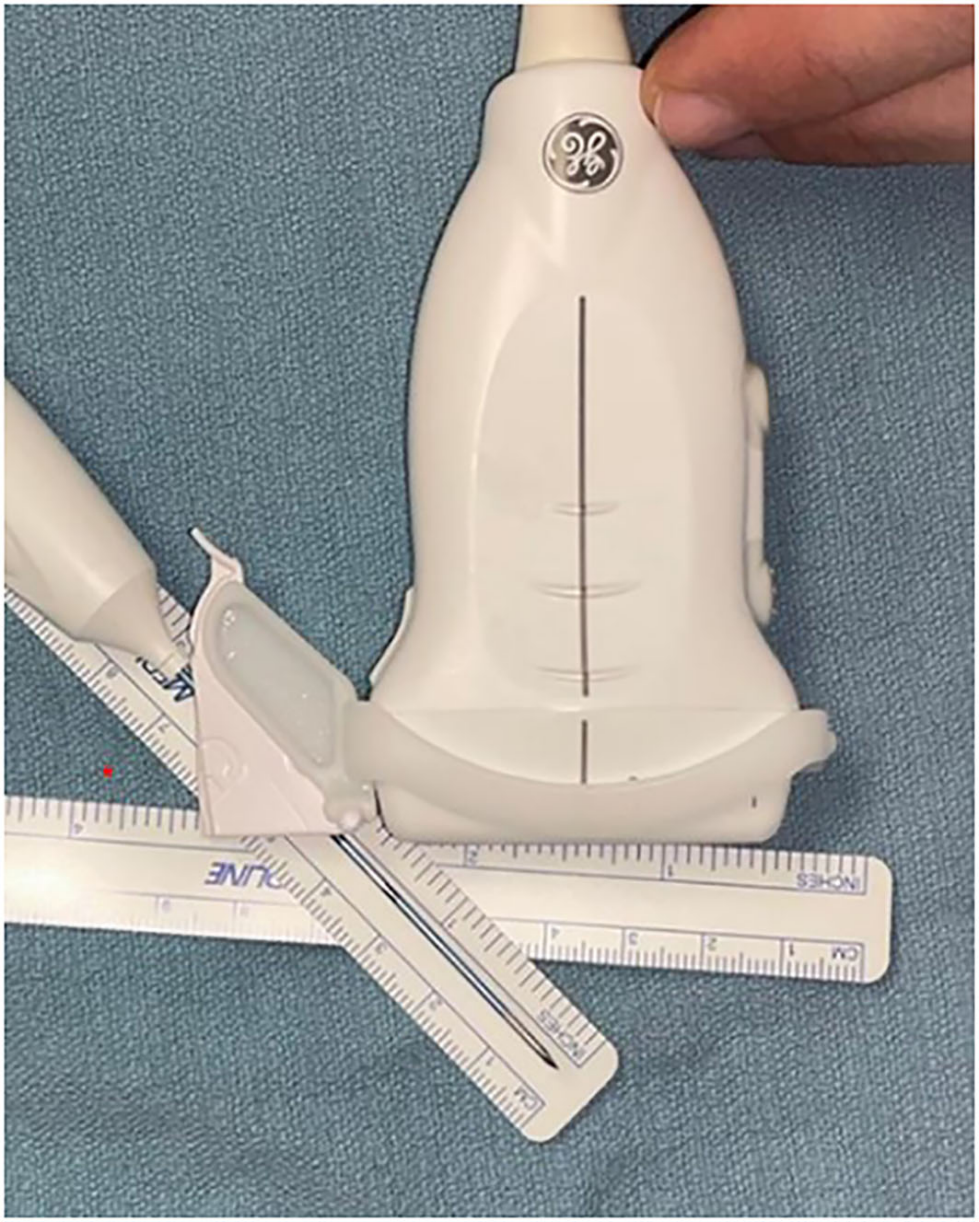
Equipment set-up The linear transducer probe guide is attached directly to an 18G radiofrequency probe guide to produce fixed translatory bodily movements with the electrode tip consistently centred in the long axis, minimizing angulation error during ablation.

A high resolution 10Mhz linear ultrasound probe is then attached to a GE Edwards ultrasound probe guide which on its lateral aspect is directly connected to an 18G fitting guide (CIVCO Infiniti Plus™ Needle Guide) for the RF probe (STARMed Seoul, Korea). (**Figures 1**, **2**) The purpose of the guide is to maintain constant inline visualisation during the procedure, removing the margin of error associated with freely holding the probe. In the results, we will demonstrate the use of this guided technique using a pictorial and geometric analysis of the probe within this guide. This will allow a greater understanding of the pros and cons of using such a guide. Research ethics board approval was not required as patients were not required for this analysis.

### Ablative technique

The moving shot technique is then implemented by the clinician whereby the nodule is divided into a series of conceptual subunits with smaller subunits at the periphery, paying close attention to the danger triangle as has been previously described (7). The radiofrequency active tip probe (STARMed Seoul, Korea) is percutaneously inserted under direct vision using the guide (**Figure 1**) via a transisthmic approach beginning at the most posterior and deepest aspect of the nodule. The primary treatment endpoint is a combination of hyperechogenic change including echogenic microbubbles on the ultrasound screen and objective monitoring of tissue impedance level. The clinician then retracts the probe along the guide, consistently visualising the probe tip, and proceeds to ablation of the next conceptual subunit until all units have developed a visualised hyperechogenic focus with high tissue impedance proceeding from deep to superficial, and sparing the subunits contained within the danger triangle.

The patient is then monitored for at least one hour following the procedure to ensure there are no complications encountered and subsequently followed up by phone call within 24 hours of the procedure to monitor for any symptom change.

## Results

**Figure 3:**
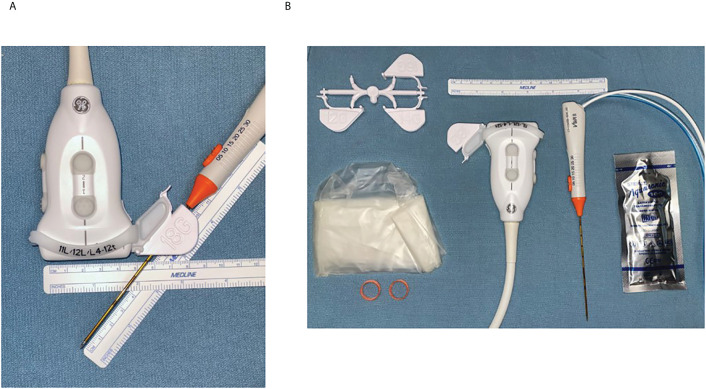
The combination of an 18G guide to maintain in line visualisation combined with a 10cm length RF probe **(A)** provides a working field of approximately 28cm^2^ whilst applying the moving shot technique. Using a 7cm RF probe, the cross-sectional area achieved is approximately 7cm^2^ but can be increased to 13cm^2^ by trimming the guide using sterile scissors as shown in **(B)**.

**Figure 4:**
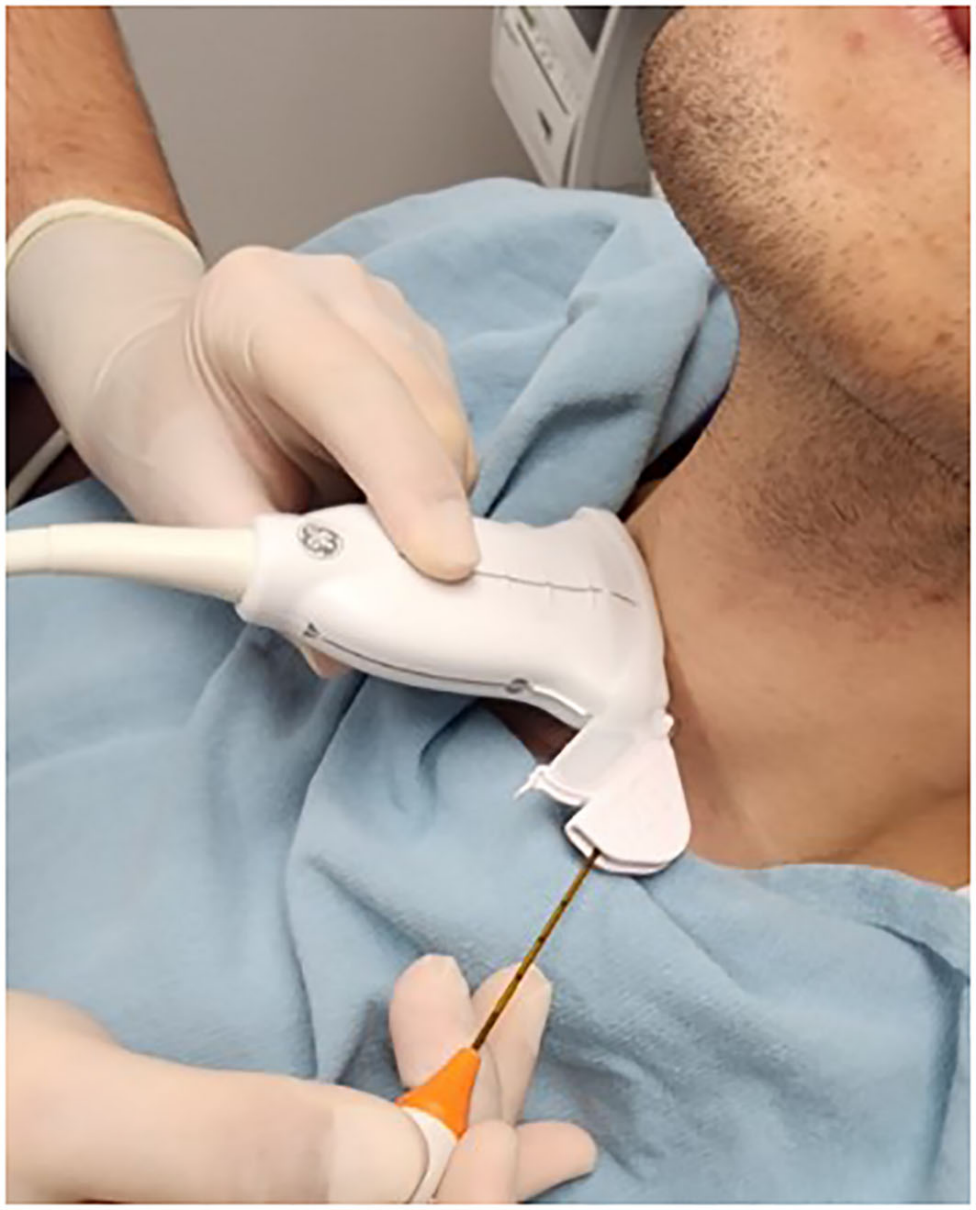
Schematic representation of the use of the RF probe guide in ablation of a 4cm thyroid nodule. A total cross sectional of 28cm^2^ can be ablated through a single puncture site whilst maintaining in line visualisation of the tip at all times.

Using the guided technique, a 7cm probe allows a maximum usable length of 3cm and cross-sectional area of 7.1cm^2^. Trimming of the guide, facilitates a further 1cm of length, resulting in a cross-sectional area of 13cm^2^. A 10cm probe provides a usable length of 6cm using the guide with a cross-sectional area attainable of 28.3cm^2^, that can be increased to 38.5cm^2^ once the guide is trimmed. Accounting for the thickness of the overlying subcutaneous tissue and skin, the guided technique can be safely used to ablate a 3cm nodule with a 7cm probe and up to a 6cm nodule with the 10cm probe once the guide is trimmed. A summary of the geometric analysis of both guides has been provided in **Table 1**. **Figures 3**, **4** provide a schematic geometric analysis using the 10cm RF probe (STARMed Seoul, Korea) length of a potential arc of treatment using a 4cm right thyroid nodule as an example with the ability to ablate a total area of 28cm^2^ using the guide with constant probe tip visualisation. The advantages and disadvantages of such an approach have been summarized in **Table 2** below.

**Table 1:** Geometric analysis comparing the 7cm RF probe to the 10cm RF probe.

	7cm RF Probe	10cm RF Probe
Cost	$	$$
Maximum usable length (without trim)	3cm	6cm
Maximal usable length (with trim)	4cm	7cm
Cross sectional area	7.1cm^2^	28.3cm^2^
Cross sectional area with trimmed guide	13cm^2^	38.5cm^2^
Nodule diameter (maximum)	3cm	6cm

**Table 2:** Summary of the advantages and disadvantages of guided RF ablation.

	Advantages	Disadvantages
**Guided**	Easier long axis visualisation of the electrode tip at all times Low cost and user friendly Attaches to almost any 10MHz ultrasound probe Simplifies electrode tip control in the long axis for the novel interventionalist	Obscures the site of puncture and therefore potentially increases the chance and need of multiple puncture sites Limits the depth of the electrode tip for large or deep nodules >5cm Additional cost to treatment May need modification for use with 7 cm probe
**Non-guided**	Has been used for several years with a proven safety profile Doesn’t require the additional purchase of a guide Allows direct visualisation of the puncture site	Susceptible to movement artefact associated with deglutition, talking and reactions to discomfort Can be difficult to maintain in-line visualisation of the probe tip at all times More challenging than the use of a guide for novel interventionalists

## Discussion

Radiofrequency ablation of the thyroid gland provides the thyroid interventionalist with a minimally invasive technique to ablate benign thyroid and AFTN in an outpatient setting, without a general anaesthetic, leaving them without a scar and most importantly, avoiding the risk of hypothyroidism. The efficacy of RFA in benign thyroid disease has been previously well established. Most recently, Russell et al. in a multi-institutional prospective collection of 620 patients from a North American population with benign thyroid nodules and a baseline nodule volume of 5.6mL, followed with serial ultrasound and TSH levels for 12 months, demonstrated a median volume reduction rate of 71% at 12 months, with 78% of patients achieving treatment success defined as a volume reduction greater than 50% at 1 year. On multivariate analysis, small nodules (with a treatment volume <10mls) and medium nodules (10-20mls) achieved a success rate of 81% and 87% at 12 months respectively (18). A similar muti-institutional prospective Korean study consisting of 276 patients demonstrated a mean volume reduction of 80% at 12 months with a mean preoperative volume of 14mls and largest diameter of 3.8cm, however, one quarter of these patients also had another ablative procedure (19). When followed by serial ultrasound for 6 years, the mean volume reductions at 24, 36, 48 and 60 months were 84%, 89%, 92% and 95% respectively. Deandrea et al. in their randomized control international collaborative trial across two centres in Italy and Korea demonstrated a mean volume rate reduction of 71% at 6 months (20), and a recent systematic review demonstrated a mean volume reduction of 67-75% at 12 months in patients treated with a single procedure, which increased to 94% in patients with repeated ablations consistent with previous findings (21). Thus, the clinical outcome of volume reduction in benign thyroid nodules has previously well documented.

RFA has also recently expanded its use in achieving not only volume reduction of thyroid nodules, but biochemical euthyroidism of patients with AFTN, where the risk of malignancy is exceedingly low. In a systematic review and meta-analysis conducted by Cesareo et al, the pooled rate of patients with TSH normalization was 57% with a pooled volume reduction rate of 79% at one year (22). The rate of success of RFA in AFTN has been significantly correlated not only with volume rate reduction but also with size of nodule, with nodules more likely to respond to treatment when baseline volume is <12mls (23). This has been postulated to relate to the ability to achieve complete ablation of conceptual subunits in smaller volume nodules, minimizing the risk of untreated autonomously hyperfunctioning residual tissue. Therefore, RFA has now been recommended as a treatment option in the majority of internationally established guidelines.8-14 Our practice is currently limited to patients with biopsy confirmed benign nodules or toxic adenomas that are less than 20mls in volume and located away from the danger triangle given the potential for undertreating hyperfunctioning ablation subunits to avoid risk of thermal injury.

Current consensus guidelines have established there is a significant learning curve for thyroid ablation required prior to providing optimal treatment for patients (8). Complete ablation of all conceptual subunits optimizes patient outcomes by increasing the success of the procedure and decreasing the need for additional treatment. The most recent ATA consensus statement indicates that clinical efficacy requires approximately 30 cases based on the results of three previously published series (24–26). We agree with the aforementioned studies and all our procedures are currently performed by two Otolaryngology Head and Neck Surgeons with significant previous expertise in both US and FNA. In addition to this, Improvements in volume rate reduction in benign thyroid nodules has also been attributed to expertise in electrode manoeuvrability, the amount of energy applied to each conceptual subunit and the use of colour doppler during the procedure to identify viable thyroid tissue (22). We feel that our novel guided technique proposed has several advantages. Firstly, the use of a radiofrequency probe guide attached to a high frequency linear ultrasound transducer ensures visualisation of the needle under direct vision for the entirety of the procedure. The probe can then be angled to target separate subunits of varying depth through the same puncture site. We have found the stability gained by the guide improves operator confidence in ablation of smaller conceptual subunits in close proximity to the recurrent laryngeal nerve and the carotid artery, areas often left unattended due to risk of injury. Secondly, the placement of a fixed guide negates the human error in movement often unavoidable during both talking and swallowing which we have found to be a significant advantage. It also facilitates ablation of multiple subunits through a single-entry site by rotating the probe in a counterclockwise fashion, commencing inferiorly and moving superiorly, yet maintaining direct vision. Further to this, the restraint of a guide facilitates the need for a systematic approach to ablation requiring the clinician to subdivide the outlined nodule into horizontal tracts spaced approximately 1cm apart, ensuring a more complete ablation. One of the challenges we recognise in the implementation of a novel interventional technique, pertains to not only familiarity with instrumentation but also the ability to consistently replicate the same result under varied conditions in patients with variable anatomy and we feel the use of a guide aids in achieving this. Further to this, we feel that in the infancy of clinicians adopting radiofrequency into their practice, such an approach facilitates consistency, adaptability and accounts for potential movement artefact otherwise experienced during deglutition, speech or as a reaction to discomfort experienced by the patient during the procedure. This is further enhanced by the use of a 10cm radiofrequency probe allowing an arc of rotation of 6cm, not otherwise achieved with shorter probes, highlighting its use in large benign nodules. However, the 7cm radiofrequency probe can also be used with modifications made to the needle guide. Of course, an individualized approach is required based on the patient’s body habitus, the size and depth of the nodule and the clinician needs to remain cognizant of potential complications including nodule rupture, hematoma, nerve and tracheal injury which remain of paramount importance.

There is a currently a large amount of literature supporting radiofrequency ablation of thyroid nodules, however this is only amongst high volume thyroid interventionalists. As the use of RFA becomes more widespread amongst surgeons, a guided approach with the electrode tip fixed within the centre of the long axis field consistently, produces translatory bodily movement of both transducer and RF probe endorsing confidence in achieving accurate subunit ablation. This will allow the thyroid interventionalists to consistently ablate more conceptual subunits and potentially improve the efficiency of RFA in the management of both benign and AFTN. Further prospective studies will aid in elucidating this as the technology increases in adoption rates across North America.

## Conclusion

We present a novel radiofrequency probe guide which is simple and easy to attach to an ultrasound probe that provides a valuable addition to the head and neck surgeons armamentarium in RFA of thyroid nodule.

## Data availability statement

The original contributions presented in the study are included in the article/supplementary material. Further inquiries can be directed to the corresponding author.

## Author contributions

LS: Writing – review & editing, Writing – original draft, Formal analysis, Data curation, Conceptualization. KH: Writing – review & editing, Writing – original draft, Validation, Methodology, Investigation, Formal analysis, Data curation, Conceptualization. DE: Writing – review & editing, Writing – original draft, Methodology, Investigation, Formal analysis, Conceptualization. AE: Writing – review & editing, Writing – original draft, Visualization, Validation, Supervision, Software, Resources, Project administration, Methodology, Investigation, Funding acquisition, Formal analysis, Data curation, Conceptualization.
